# Impact of Precision Medicine on Clinical Outcomes: A Single-Institution Retrospective Study

**DOI:** 10.3389/fonc.2021.659113

**Published:** 2021-08-31

**Authors:** Ryann Quinn, Rajvi Patel, Cristina Sison, Amandeep Singh, Xin-Hua Zhu

**Affiliations:** ^1^Northwell Health Cancer Institute, New Hyde Park, NY, United States; ^2^Biostatistics Unit, Feinstein Institutes for Medical Research, Northwell Health, Manhasset, NY, United States; ^3^Donald and Barbara Zucker School of Medicine at Hofstra/Northwell, Hempstead, NY, United States

**Keywords:** precision medicine, FoundationOne, next-generation sequencing, precision oncology, targeted treatment approaches

## Abstract

**Purpose:**

The strategy of precision medicine has been widely adopted in the practice of oncology, although the efficacy remains unclear. This study assesses clinical outcomes in patients with an actionable alteration found during FoundationOne CDx™ (F1CDx) testing and who received a targeted therapy based on the results.

**Materials and Methods:**

This is a retrospective cohort study of patients with tumors that underwent F1CDx from September 2012 to July 2018. F1CDx provided actionable alterations for patients to select appropriate therapies. The primary objective was to estimate the objective response rate (ORR) at 3 months from the start of study treatment. The secondary objectives were to estimate progression-free survival (PFS) and overall survival (OS).

**Results:**

One thousand patients underwent F1CDx testing. Six hundred fifty-two patients were identified as having actionable mutations. Thirty-eight patients (18 males and 20 females) received targeted therapy and were included in the study. The most common alterations were PD-1/PDL-1, high-TMB, P13K, and HER2/ERBB2. Patients received various treatments including nivolumab, pembrolizumab, trastuzumab, and everolimus. Eight (23.5%) and six (17.7%) patients achieved partial response (PR) and stable disease (SD), respectively; 20 (58.8%) had progression of disease (PD). The disease control rate was 41.2% (95% CI: 24.7% to 59.3%). The median PFS was 2.7 months (95% CI: 2.3 to 5.4 months), and median OS was 9.9 months (95% CI: 4.5 to 33.7 months).

**Conclusion:**

Our results demonstrate promising data in precision medicine in real community oncology practice. It warrants further large and prospective studies in patients with actionable alterations.

## Introduction

Advances in genomics and next-generation sequencing (NGS) have revolutionized the field of oncology by allowing precision-based approaches in management of cancer. Precision oncology looks at genetic and molecular characteristics of tumors instead of traditional histology to match treatment strategies (1). This strategy has been applied in the management of many types of cancers including testing epidermal growth factor receptor (EGFR) mutation, anaplastic lymphoma kinase (ALK) fusion, B-type Raf kinase (BRAF) mutation, c-Ros oncogene 1 (ROS1), and programmed death ligand-1 (PD-L1) in nonsmall-cell lung cancer (NSCLC); human epidermal growth factor receptor 2 (HER2) in breast and gastric cancer; Kirsten rat sarcoma viral oncogene homolog (K-RAS), neuroblastoma ras viral oncogene homolog (N-RAS), and BRAF mutation in colon cancer; and tumor mutational burden, NTRK fusions, and microsatellite instability (MSI) status in all solid tumors (2–8). Biomarker-matched targeted therapies for specific alterations in approximately 40 different cancer genes are available that are Food and Drug Administration (FDA) approved (9, 10). In addition, many precision clinical trials in oncology which aim to determine if targetable gene alterations can predict response to targeted therapies were done and are currently being conducted. Results from selected arms of the National Cancer Institute-Molecular Analysis for Therapy Choice (NCI-MATCH) trial are available and have mixed results. While the BRAF V600E/K mutation cohort (dabrafenib and trametinib) and the MSI-H cohort (nivolumab) had positive results, other arms such as the fibroblast growth factor receptor (FGFR) alteration cohort (FGFR inhibitor AZD4547), the PIK3CA-mutation cohort (taselisib), and the HER2 amplification cohort (ado-trastuzumab emtansine) had negative results (11–15). The biomarkers PDL-1 and tumor mutation burden (TMB) have been shown to predict responses to immunotherapy in several cancers and are also included in our study as “targeted treatment”.

The efficacy of precision oncology remains unclear. However, the strategy of precision medicine has been widely adopted in the practice of community oncology. The FoundationOne CDx™ test (F1CDx™) was the first NGS-based gene panel to be approved by the FDA for the detection of alterations that may confer benefit from FDA-approved treatments for certain cancers (16). Two additional panels (Memorial Sloan Kettering (MSK)-IMPACT and Oncomine Dx Target Test) have subsequently been approved to detect tumor gene alterations in certain cancer types (17). At our institution, we frequently order F1CDx testing on patients with advanced cancer resistant to standard of care, or in rare cancers in which there is no standard of care. It is necessary to report the efficacy and problems such as low treatment rate of precision medicine in the real world among patients with multiple targetable alterations and multiple cancer types. We conducted a retrospective cohort study of patients treated at our institution that received nonstandard-of-care–targeted therapy based on the results of F1CDx testing. This study examines our experience with clinical outcomes in patients identified as having an actionable alteration through F1CDx testing and who received a targeted therapy based on the results.

## Materials and Methods

We performed a retrospective cohort study of patients with tumors that underwent F1CDx from September 2012 to July 2018 at the Northwell Health Monter Cancer Center. The study was approved by our institutional review board. Patients were identified using the Foundation Medicine patient reports database. Patients were excluded if they were less than 18 years of age or if F1CDx testing was done for a hematologic malignancy. Patients’ age, diagnosis, ethnicity, number of prior treatments, actionable alterations identified by F1CDx testing, and available targeted treatments were recorded. We also calculated the growth modulation index (GMI) for each patient that had a prior line of therapy by calculating the ratio of progression-free survival (PFS) on targeted therapy (PFS*_n_*) *vs*. PFS on prior line of treatment (PFS*n*-1).We used the electronic medical record to determine if patients received the targeted treatment suggested by the F1CDx report; if they did not receive targeted treatment, we recorded the next treatment received after F1CDx testing if available. We excluded patients who received targeted therapy that was FDA approved and standard of care for the patient’s primary tumor site. Of note, patients with MSI-H and tumor mutational burden included in this study received targeted treatments prior to FDA approval dates.

### Statistical Considerations

The primary objective of the study was to estimate the objective response rate (ORR) at 3 months from the start of study treatment. ORR was defined by imaging findings as per Response Evaluation Criteria in Solid Tumors version 1.1 (RECIST v1.1) (18). Secondary objectives were to estimate PFS and overall survival (OS).

Descriptive statistics (frequencies, proportions, means, standard deviations, medians, IQR) were calculated to describe the demographic and clinical characteristics. The ORR at 3 months from start of treatment was estimated using standard methods for estimating proportions and their associated 95% confidence intervals. The Kaplan-Meier product limit method was used to estimate PFS and OS. PFS was measured from the time of initiation of targeted treatment therapy to the first documentation of disease progression or death due to any cause. Patients who have not achieved the event of interest (progression or death for PFS and death for OS) as of the last documented follow-up were considered “censored” and the last follow-up time was used in the analysis.

### Sample Size Considerations

While there were 652 patients with actionable mutations in the F1CDx database, only 38 patients (5.8%) received targeted therapy based on the results and were included in the study. Thus, the total sample size for this study was 38 subjects. The sample size was a sample size of convenience and was based on feasibility and availability of subjects that meet inclusion criteria; it was not based on any formal statistical power calculations.

## Results

**Figure 1:**
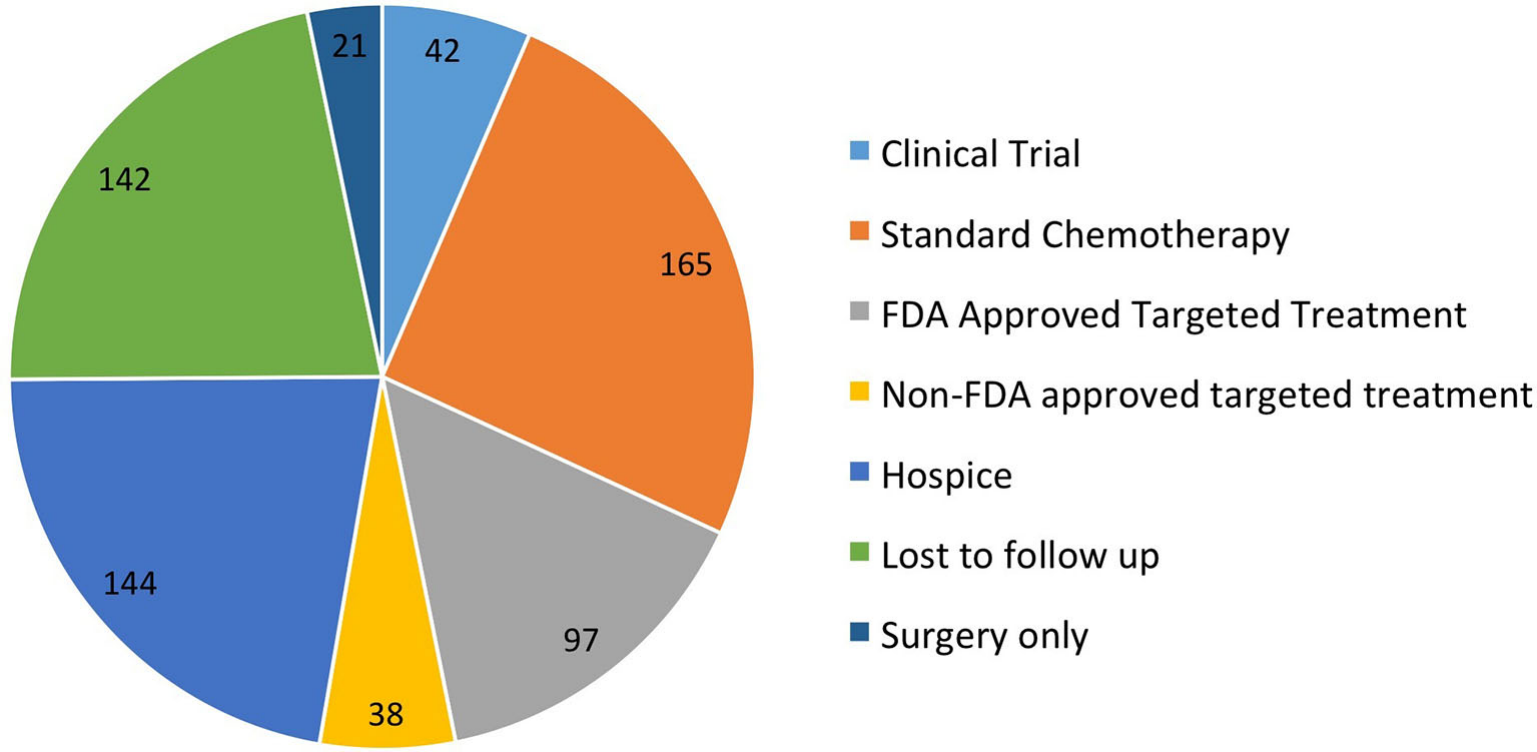
Treatment received after results of F1CDx testing available.

One thousand patients at our institution had tumor samples sent for F1CDx testing from September 2012 to July 2018. Six hundred fifty-two patients had actionable alterations with available targeted treatments. Of the 652 patients, 42 (6.4%) went on a clinical trial, 165 (25%) received standard next-line chemotherapy, 135 (20.7%) received targeted therapy, 144 either went on hospice or died prior to receiving treatment (22%), 142 were lost to follow-up (22%), 21 were treated with surgery only (3.2%), and three (0.04%) had issues with insurance approval of targeted therapy (**Figure 1**). Of the 135 patients that were treated with targeted therapy, 97 received FDA approved targeted therapy (15%) and 38 (5.7%) received non-FDA-approved targeted therapy. The 38 patients that received non-FDA-approved targeted therapy were included in the analysis.

**Figure 2:**
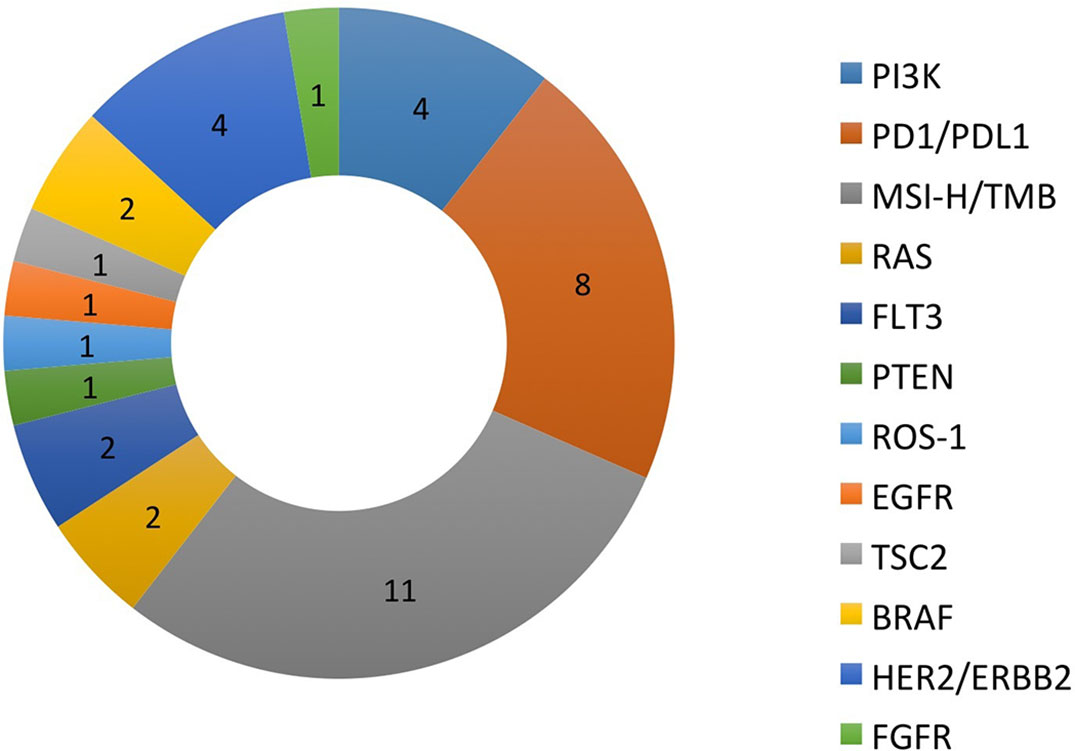
Target gene and frequency.

**Figure 3:**
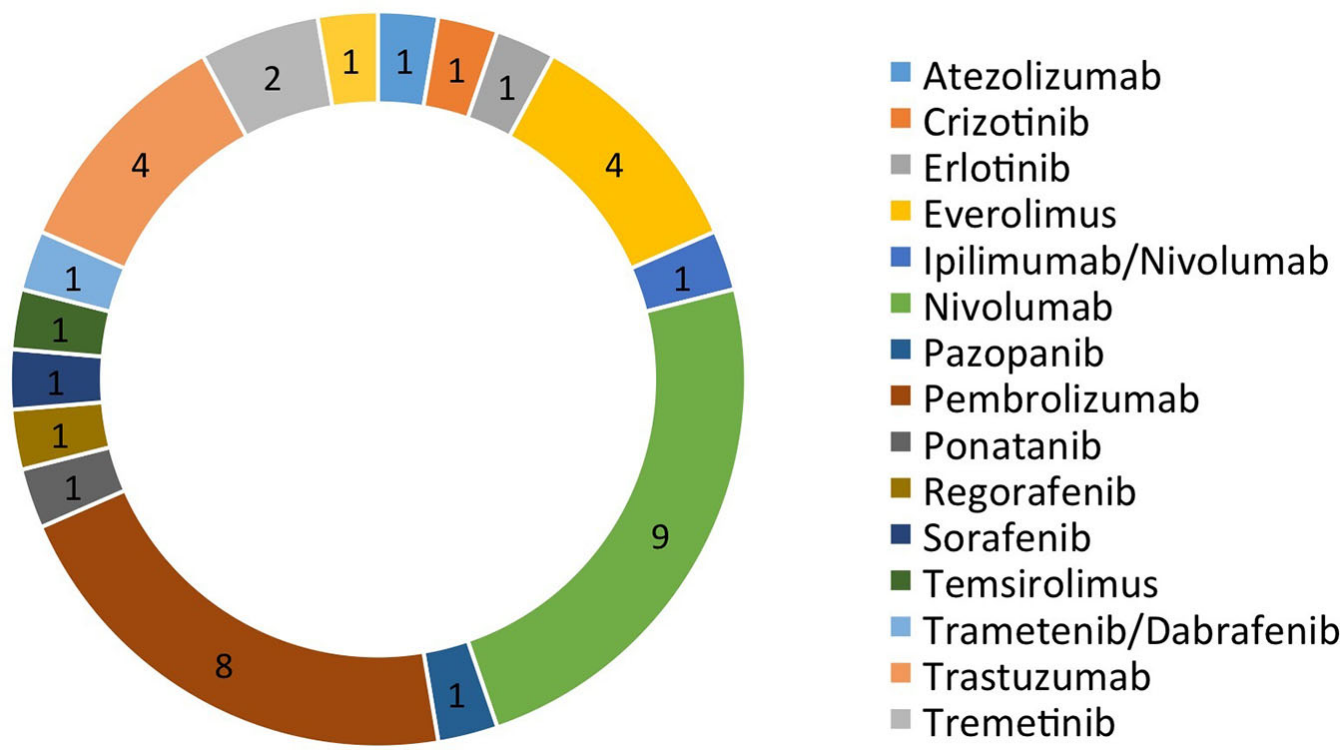
Targeted treatment received and frequency.

There were a total of 38 patients (18 males and 20 females) in the study sample (**Table 1**). Mean age of patients was 57.7 years, ranging from 20 to 92 years. The majority (35/38 = 92.1%) had at least one other prior systemic therapy. Patients included in the study had tumors with various targetable alterations (**Figure 2**). The most common alterations were the PD-1/PDL-1 (eight patients, 21.1%) and high-TMB (eight patients, 21.1%). There were four patients with P13K gene (10.5%) and four patients with HER2/ERBB2 (10.5%). Patients were diagnosed with various types of primary tumors including breast, gastrointestinal, head and neck, lung, neuroendocrine, ovarian, urothelial, and unknown primary adenocarcinoma (**Table 2**). There were nine patients with colon adenocarcinoma, five patients with unknown primary adenocarcinoma, and three patients with urothelial cell carcinoma. Patients received various targeted treatments including nivolumab (nine patients; 23.7%), pembrolizumab (eight patients; 21.1%), trastuzumab (four patients; 10.5%), and everolimus (four patients; 10.5%); treatment distribution of the rest of the 13 patients is shown in **Figure 3**. The molecular alterations and matched treatment for each patient is listed in **Table 3**. The average time from F1CDx testing to receiving targeted treatment was 184 days. The median time was 97 days.

**Table 1:** Patient characteristics.

Characteristic	No. of patients (%)
Sex	
Female	20 (53%)
Male	18 (47%)
Median age [years (range)]	58 (20–92)
Race/ethnicity	
White	21 (55%)
Black	10 (26%)
Asian	4 (11%)
Hispanic	2 (5%)
Declined	1 (3%)
Prior lines of therapy	
0	3 (8%)
1	8 (21%)
2	10 (26%)
3	11 (29%)
>3	6 (16%)

**Table 2:** Tumor histology.

Tumor histology	Frequency
Breast	
Breast invasive ductal carcinoma	1
Breast angiosarcoma	1
Gastrointestinal	
Colon adenocarcinoma	9
Other gastrointestinal adenocarcinoma	7
Lung	
Lung adenocarcinoma	1
Genitourinary	
Kidney epitheliod angiomyolipoma	1
Urothelial cell carcinoma	3
Gynecologic	
Cervical squamous cell carcinoma	1
Ovarian adenocarcinoma	1
Uterine carcinosarcoma	1
Head and neck	
Squamous cell carcinoma	1
Thymic carcinoma	1
Neuroendocrine	
Neuroendocrine carcinoma	3
Skin	
Squamous cell carcinoma	1
Unknown primary	
Unknown primary adenocarcinoma	5
Unknown primary squamous cell carcinoma	1

**Table 3:** Molecular alteration and matched therapy.

Patient	Diagnosis	Molecular alteration	Matched therapy
1	Urothelial cell carcinoma	PI3K	Temsirolimus
2	Colon adenocarcinoma	High TMB	Pembrolizumab
3	Colon adenocarcinoma	High TMB	Nivolumab
4	Esophageal adenocarcinoma	PTEN	Everolimus
5	Small intestine adenocarcinoma	ROS-1	Crizotinib
6	Colon adenocarcinoma	MSI-H	Pembrolizumab
7	Neuroendocrine carcinoma (rectal)	PDL-1	Nivolumab
8	Unknown primary adenocarcinoma	PDL-1	Nivolumab
9	Cervix-squamous cell carcinoma	PI3K	Everolimus
10	Skin squamous cell carcinoma	MSI-H	Nivolumab
11	Duodenal adenocarcinoma	MSI-H	Nivolumab
12	Head and neck squamous cell carcinoma	RAS	Trametinib
13	Unknown primary adenocarcinoma	EGFR	Erlotinib
14	Neuroendocrine lung carcinoma	PDL-1	Nivolumab
15	Kidney epithelioid angiomyolipoma	TSC2	Everolimus
16	Colon adeocarcinoma	FLT3	Ponatanib
17	Lung adenocarcinoma	BRAF	Vemurafenib
18	Colon adenocarcinoma	RAS	Tremetinib
19	Breast angiosarcoma	PI3K	Sorafenib
20	Colon adenocarcinoma	HER2/ERBB2	Trastuzumab
21	Unknown primary adenocarcinoma	HER2/ERBB2	Trastuzumab
22	Thymic carcinoma	PDL-1	Pembrolizumab
23	Unknown primary adenocarcinoma	PDL-1	Nivolumab
24	Rectal adenocarcinoma	High TMB	Pembrolizumab
25	NET with small cell features	High TMB	Ipilimumab/Nivolumab
26	Urothelial carcinoma	PI3K	Everolimus
27	Esophageal adenosquamous carcinoma	High TMB	Pembrolizumab
28	Breast cancer	High TMB	Pembrolizumab
29	Pancreatic adenocarcinoma	HER2/ERBB2	Trastuzumab
30	Colon adenocarcinoma	HER2/ERBB2	Trastuzumab
31	Ovarian cancer	PDL-1	Pembrolizumab
32	Uterine carcinosarcoma	FGFR	Pazopanib
33	Gastric adenocarcinoma	PDL-1	Pembrolizumab
34	Unknown primary squamous cell carcinoma	High TMB	Nivolumab
35	Urothelial carcinoma	High TMB	Atezolizumab
36	Unknown primary adenocarcinoma	PDL-1	Nivolumab
37	Colon adenocarcinoma	FLT3	Regorafenib
38	Colon adenocarcinoma	BRAF	Trametenib/Dabrafenib

Of the 1,000 total patients tested, the most common alteration was KRAS (200 patients, 20%), followed by PI3K gene alterations (65 patients, 6.5%) and BRAF alterations (60 patients, 6%). Prevalence of the 15 most common gene alterations found in >10 patients is shown in **Table 4**.

**Table 4:** Prevalence of actionable alterations among 1,000 patients studied.

Alteration	Number of patients	Percentage (%)
KRAS	200	20
PIK3CA	65	6.5
BRAF	60	6
TMB	57	5.7
ERBB2/HER2	55	5.5
STK11	52	5.2
EGFR	32	3.2
PDL-1	31	3.1
FGFR1	29	2.9
FLT3	26	2.6
CCND1	25	2.5
BRCA2	18	1.8
NF2	16	1.6
KIT	16	1.6
PDGFRA	11	1.1

Of the 38 patients, 34 (89.5%) had a first interval scan performed from 1 to 5 months after start of targeted therapy; 13 of the 38 patients (34.2%) had a second interval scan performed between 4 to 9 months after initiation of targeted treatment therapy; and seven of the 38 patients (18.4%) had a third interval scan performed between 10 and 16 months after the start of targeted treatment therapy. Follow-up times ranged from 0.8 to 16.1 months. The median follow-up time was 2.7 months (mean, 5.1 months, SD = 4.3 months).

**Figure 4:**
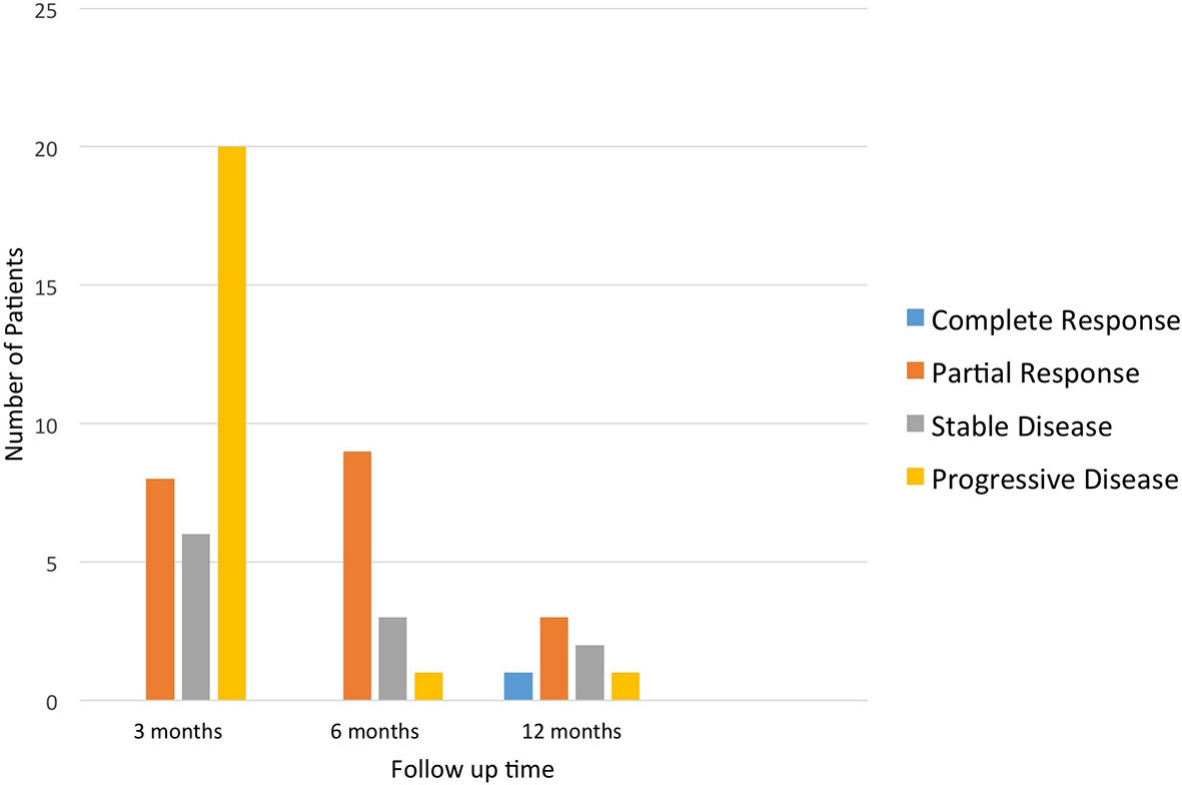
Overall response rate of patients who received targeted treatment at different time points.

Of the 34 patients evaluated for their ORR at the first scan, there were eight (23.5%) and six (17.7%) with PR and SD, respectively; 20 (58.8%) had PD. Of the 13 patients with available data at the second scan, nine (69.2%) and three (23.1%) had PR and SD, respectively; one (7.7%) had PD. Of the seven patients with available data at the third scan, one (14.3%), three (42.9%), and two (28.6%) had CR, PR, and SD, respectively; one (14.3%) had PD (**Figure 4**). Note that for the second and third interval scans, the number of evaluable observations decreased substantially due to patients having progression of disease or death after the first interval scan or not yet reaching the timepoint for the 2nd or 3rd interval scans. The interpretation of the proportions of ORR and favorable response rates for the second and third interval scans should be interpreted with caution due to potential missing information.

**Figure 5:**
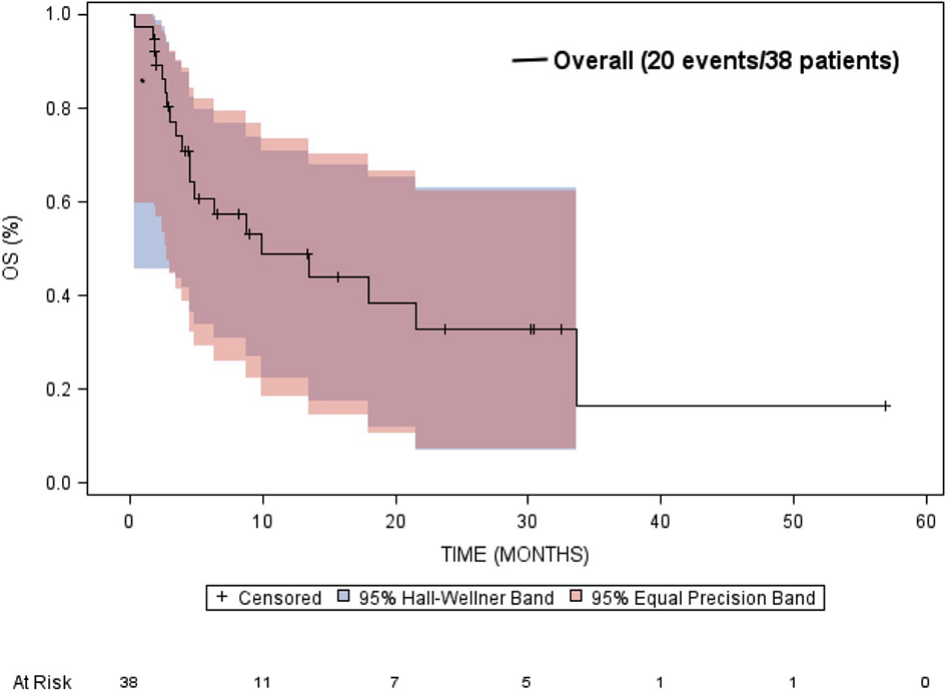
Kaplan-Meier estimated overall survival (OS) in patients who received targeted treatment.

**Figure 6:**
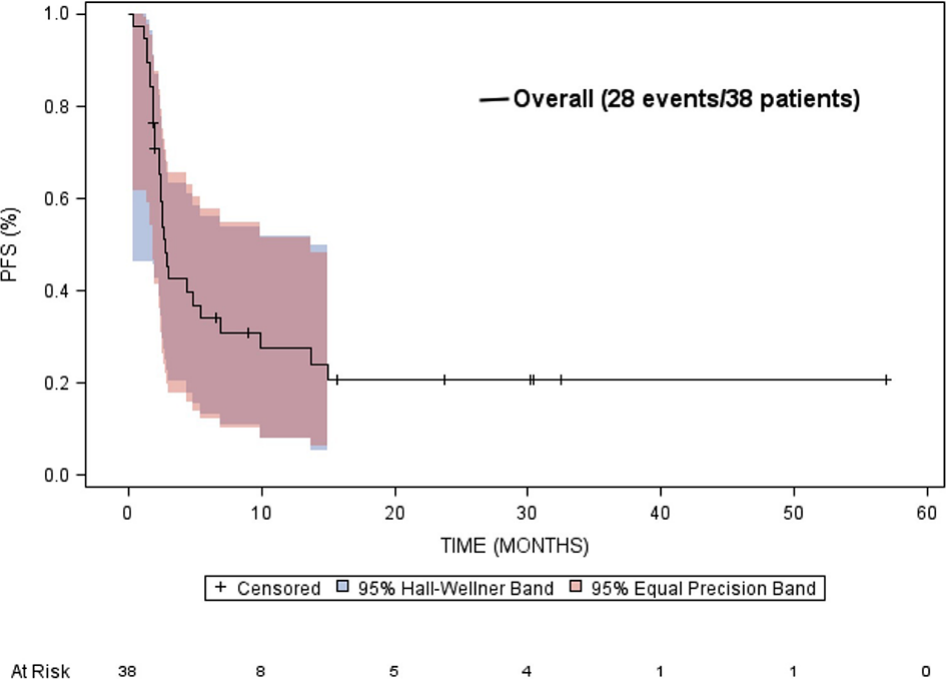
Kaplan-Meier estimated progression free survival (PFS) in patients treated with targeted therapy.

The median overall survival was estimated to be 9.9 months (95% CI: 4.5 to 33.7 months) (**Figure 5**). Neither gender or age at diagnosis was associated with overall survival. The median time-to-progression was estimated to be 2.7 months (95% CI: 2.3 to 5.4 months) (**Figure 6**). Neither gender or age at diagnosis was associated with progression-free survival.

Nine (27%) out of 33 patients who were eligible for PFS ratio analysis demonstrated a PFS ratio of ≥1.3 with exact binomial of 95% and confidence interval of 13% to 46% (**Table 5**). We did not carry out a formal statistical test since we did not prespecify a hypothesis regarding the proportion of subjects with a PFS ≥1.3.

**Table 5:** Treatment regimen received by nine patients with ratio of PFS ≥1.3.

Patient	Diagnosis	Previous regimen prior to target treatment	Target treatment received
6	Colon adenocarcinoma	Fluorouracil + oxaliplatin	Pembrolizumab
7	Neuroendocrine carcinoma (rectal)	Capecitabine + oxaliplatin	Nivolumab
8	Unknown primary adenocarcinoma	Fluorouracil + oxaliplatin	Nivolumab
11	Duodenal adenocarcinoma	Fluorouracil + oxaliplatin	Nivolumab
14	Neuroendocrine lung carcinoma	Carboplatin + etoposide	Nivolumab
17	Lung adenocarcinoma	Carboplatin + pemetrexate	Vemurafenib
18	Colon adenocarcinoma	Irinotecan + regorafenib	Trametinib
26	Urothelial carcinoma	Atezolizumab	Everolimus
38	Colon adenocarcinoma	Regorafenib	Trametinib/Dabrafenib

## Discussion

In this study, eight of 34 (23.5%) patients who had a 3-month interval scan had a partial response. Of these eight patients, three had a PDL-1 overexpression, two had a BRAF mutation, and three had sustained PR for >1 year; these patients all received immunotherapy for microsatellite instability (MSI-high). Two patients received nivolumab and one patient received pembrolizumab. One patient had a sustained CR for >1 year; this patient had lung adenocarcinoma and received vemurafenib for a BRAF mutation. Six (17.6%) patients showed stable disease. Disease control rate including partial response and stable disease was 41.1%. Even if we remove the three cases of MSI-high patients based on current standard of care, five of 31 (16.1%) with partial response and six of 31 with stable disease (19.3%), thus disease control rate was 35.4%. Although our study included multiple targetable genes and multiple cancer types which reflects the real practice of precision medicine in community oncology, and was retrospective, the objective response rate was greater than 16% in either the above-mentioned analysis. This threshold was set as a promising tumor shrinkage in the NCI-MATCH trial.

In this study, 1,000 patients had F1CDx testing and 652 patients had tumors that had actionable alterations. Of these 652 patients, 42 (6.4%) went on a clinical trial, 135 (20.7%) received targeted therapy, 97 received FDA-approved targeted therapy (15%), and 38 (5.8%) received non-FDA-approved targeted therapy. The low rate of patients receiving targeted therapy even in the presence of actionable alterations has also been seen in other studies (19–24). In the MOSCATO-1 trial, of the 411 tumors which were found to have potentially actionable alterations, only 199 (48%) were paired with a targeted therapy (19). The patients on these studies had similar reasons for not receiving targeted treatment, such as, rapid clinical deterioration, enrolling on a different clinical trial, or being lost to follow-up. At our center, as well as in other trials, a major factor preventing patients from receiving targeted therapy was decline in performance status as well as patients electing hospice rather than further therapy. This highlights the importance of shared decision making, which takes a patient’s values, performance status, and time to testing results prior to requesting NGS testing. On the other hand, one challenge with multigene NGS sequencing is that providers may have a challenge selecting the “best option” when multiple targetable drugs are available for an actionable alteration. F1CDx reported many variants of uncertain significance or equivocal copy number alterations, further complicating the selection. A recent study at an academic medical center showed that 22% of physicians reported low confidence in their knowledge of genetics (25). One study of 5,688 patients with advanced NSCLC who were treated in the community setting showed that of the 873 patients who received broad panel NGS testing, less than 5% of patients received nonapproved targeted treatment based on the results (26). The development of regional molecular tumor boards including geneticists and bioinformaticists may provide improved identification of potential off-label or investigational therapeutic options as well as assist with navigation of insurance authorization (27). Our center does not have a molecular tumor board which could have contributed to the low treatment rate. There was low enrollment of 42 patients (6.4%) on precision medicine trials.

Twenty-five percent of patients at our center who had F1CDx testing done received the next-line standard-of-care treatment. Given that broad NGS testing is unlikely to affect first-line treatment, oncologists should consider waiting until the patient relapses or is refractory to standard-of-care treatments before sending a tumor sample for NGS testing. The genetic makeup of a patient’s tumor frequently changes during the course of the disease due to intratumor heterogeneity and is often responsible for development of resistance to chemotherapy or targeted therapy. Therefore, it may be more prudent to wait until the patient progresses through standard-of-care therapies prior to sending F1CDx. This could have caused the poor response rate we saw in our study, as many of our patients were treated with targeted therapy several months or years after F1CDx testing was done. Another consideration is that current Medicare reimbursement guidelines state that Medicare will only pay for F1CDx one time per each malignancy, so the treating physician will be unable to send a repeat F1CDx panel at a later date without a large cost to the patient (28). In our study, the average time between when F1CDx testing was done and when patients received targeted treatment was 184 days, the median time was 97 days. Therefore, many patients had another line of treatment in between when the testing was done and when they received targeted treatment. It is possible that the genetic makeup of the tumor could have changed during that period.

Limitations of the study include that it was a single-center retrospective study and the small sample size. Given the small sample size, multiple different targetable alterations and tumor types were included and analyzed together, which limits the ability to analyze the efficacy of each individual targetable alteration. Given the 2.7 months median PFS, a significant proportion of patients progressed after the first scan. Larger prospective randomized controlled trials which focus on an individual gene alteration tested across multiple tumor types are needed to see if targeted therapies can improve response rates.

The GMI was developed by Von Hoff as a way to objectively evaluate PFS in studies with no control arm and in which there is no consensual standard of care treatment that can be used for comparison (29). It compares the time to progression on the study drug (PFS*n*) with the PFS with the prior line of therapy (PFS*n*_−1_), and the ratio (PFS*n*)/(PFS*n*_−1_) is calculated, therefore using each patient as their own control. Using the assumption that PFS becomes shorter with each successive line of treatment, a conservative ratio of >1.3 has been used to signify drug efficacy. In our study, nine of 33 patients (27%) achieved this ratio. Given that our study was a single-arm trial with multiple different targeted treatments used and no control group, we thought this could be a reasonable measure of efficacy.

We found 23.5% patients with partial response rate, 17.6% patients with stable disease. Disease control rate was 41.1% for the patients who received targeted treatment based on results of F1CDx testing. Our results demonstrate promising data in precision medicine in real-community oncology practice. It warrants further large and prospective studies in patients with actionable alterations. Given the low treatment rate, the experience at our cancer center recommends patient selection with good performance status and proper timing for submitting NGS. There is high demand for high-quality regional molecular tumor boards to improve the selection of targetable gene alterations. Making more oncologists aware of precision medicine trials will also help improve patients’ treatment rate. In our study, 1,000 patients received F1CDx testing and only 14 patients (1.4%) received benefit from targeted treatment. Larger studies are needed to identify the proportion of patients that will benefit from precision medicine, especially given the high cost of the testing. The use of precision medicine is rapidly increasing, our future direction is to include more patients so that we can analyze actionable alterations separately, similar to the NCI-MATCH trial.

## Data Availability Statement

The raw data supporting the conclusions of this article will be made available by the authors, without undue reservation.

## Ethics Statement

The studies involving human participants were reviewed and approved by Northwell Health IRB. Written informed consent for participation was not required for this study in accordance with the national legislation and the institutional requirements.

## Author Contributions

All authors listed have made a substantial, direct, and intellectual contribution to the work and approved it for publication.

## Conflict of Interest

The authors declare that the research was conducted in the absence of any commercial or financial relationships that could be construed as a potential conflict of interest.

## Publisher’s Note

All claims expressed in this article are solely those of the authors and do not necessarily represent those of their affiliated organizations, or those of the publisher, the editors and the reviewers. Any product that may be evaluated in this article, or claim that may be made by its manufacturer, is not guaranteed or endorsed by the publisher.
